# The “Road” to Malignant Transformation from Endometriosis to Endometriosis-Associated Ovarian Cancers (EAOCs): An mTOR-Centred Review

**DOI:** 10.3390/cancers16112160

**Published:** 2024-06-06

**Authors:** Radwa Hablase, Ioannis Kyrou, Harpal Randeva, Emmanouil Karteris, Jayanta Chatterjee

**Affiliations:** 1 College of Health, Medicine and Life Sciences, Brunel University London, Uxbridge UB83PH, UK; radwa.hablase2@nhs.net (R.H.); emmanouil.karteris@brunel.ac.uk (E.K.); 2 Academic Department of Gynaecological Oncology, Royal Surrey NHS Foundation Trust Hospital, Guildford GU2 7XX, UK; 3 Warwickshire Institute for the Study of Diabetes, Endocrinology and Metabolism (WISDEM), University Hospitals Coventry and Warwickshire NHS Trust, Coventry CV2 2DX, UKharpal.randeva@uhcw.nhs.uk (H.R.); 4 Warwick Medical School, University of Warwick, Coventry CV4 7AL, UK; 5 Institute for Cardiometabolic Medicine, University Hospitals Coventry and Warwickshire NHS Trust, Coventry CV2 2DX, UK; 6 Centre for Sport, Exercise and Life Sciences, Research Institute for Health & Wellbeing, Coventry University, Coventry CV1 5FB, UK; 7 Aston Medical School, College of Health and Life Sciences, Aston University, Birmingham B4 7ET, UK; 8 College of Health, Psychology and Social Care, University of Derby, Derby DE22 1GB, UK; 9 Laboratory of Dietetics and Quality of Life, Department of Food Science and Human Nutrition, School of Food and Nutritional Sciences, Agricultural University of Athens, 11855 Athens, Greece

**Keywords:** ovarian cancer, mTOR, endometriosis, endometrioid ovarian carcinoma, clear-cell carcinoma, mTOR inhibitors

## Abstract

**Simple Summary:**

Ovarian cancer is the eighth most common cancer among women globally and 207,000 women die every year, whereas endometriosis affects around 10% of women of reproductive age. Endometriosis-associated ovarian cancers (EAOCs) frequently arise from ectopic endometrium (i.e., the presence of endometrial/stromal cells outside the uterine cavity) in the ovary. Over the past decades, there has been an increasing volume of evidence to suggest that signalling centred around the mechanistic target of rapamycin (mTOR) plays an important role in cellular functions such as proliferation, survival, and autophagy. This review summarizes the current landscape of mTOR signalling in these gynaecological malignancies and the emerging therapeutic options.

**Abstract:**

Ovarian cancer is an umbrella term covering a number of distinct subtypes. Endometrioid and clear-cell ovarian carcinoma are endometriosis-associated ovarian cancers (EAOCs) frequently arising from ectopic endometrium in the ovary. The mechanistic target of rapamycin (mTOR) is a crucial regulator of cellular homeostasis and is dysregulated in both endometriosis and endometriosis-associated ovarian cancer, potentially favouring carcinogenesis across a spectrum from benign disease with cancer-like characteristics, through an atypical phase, to frank malignancy. In this review, we focus on mTOR dysregulation in endometriosis and EAOCs, investigating cancer driver gene mutations and their potential interaction with the mTOR pathway. Additionally, we explore the complex pathogenesis of transformation, considering environmental, hormonal, and epigenetic factors. We then discuss postmenopausal endometriosis pathogenesis and propensity for malignant transformation. Finally, we summarize the current advancements in mTOR-targeted therapeutics for endometriosis and EAOCs.

## 1. Introduction

Ovarian cancer is a heterogeneous disease covering a broad range of subtypes and including peritoneal and fallopian tube tumours [1]. The World Health Organization (WHO) and the International Federation of Gynecology and Obstetrics (FIGO), in their early reports, categorized ovarian cancer based on microscopic appearance and morphological features [2,3]. Of these, the most prevalent is ovarian carcinoma, a term synonymous for epithelial ovarian cancers (EOCs). To date, the morphological appearance remains the mainstay of EOCs’ sub-classification, with several histopathological types falling under EOCs, including high-grade serous (HGSC, 70%), endometrioid (EC, 10%), clear-cell (CCC, 6–10%), low-grade serous (LGSC, 5%), and mucinous carcinoma (MC, 3–4%) [4,5]. Each of these has a different precursor lesion, prognosis and biological behaviour [6,7,8]. Of note, the precursor lesions of endometrioid and clear-cell adenocarcinoma have been linked to endometriosis and are collectively described as endometriosis-associated ovarian carcinomas (EAOCs) [6,7,8].

Endometriosis is a benign, inflammatory condition characterized by the presence of functional endometrial glands outside the uterine cavity [9,10]. It mainly affects women in their reproductive years, with a prevalence of 5–10%. Symptoms may include dysmenorrhea, chronic pelvic pain, dyspareunia, dyschezia, and infertility [11]. Although less common, endometriosis has been reported in 2–4% of postmenopausal women [12]. Overlapping symptoms with various other conditions and the absence of symptoms in some women suggest that these figures may potentially be an underrepresentation of the true incidence of the disease [13]. Several hypotheses have been suggested to explain the pathogenesis of endometriosis. The most widely accepted theory, Sampson’s retrograde menstruation, stipulates that endometriosis originates from a retrograde reflux of viable endometrial tissue into the peritoneal cavity during menstruation [9,10]. Whilst this theory may explain endometriosis in premenopausal women, it remains uncertain whether endometriosis in postmenopausal women represents a continuation of premenopausal endometriosis or arises as a “de novo” development [14]. Hormonal replacement therapy (HRT) can potentially increase the risk of endometriosis reactivation in this age group [15].

The interaction of the endometrial cells with the surrounding microenvironment at the ectopic locations modulates their cellular response. Eventually, endometriotic cells acquire cancer-like characteristics such as increased cellular invasiveness and adhesiveness, resistance to cell death, altered immune function and metabolic reprogramming [10]. These survival capabilities enable endometriosis to implant, grow, metastasize, and invade other tissues [10]. The association between endometriosis and ovarian cancer has been an area of extensive research. Several clinical and histological findings have reported the co-existence of endometriosis with clear-cell or endometrioid carcinomas [16]. Notably, endometriosis is not a pre-cancerous condition but a benign disease with malignant propensity. Indeed, atypical endometriosis has been observed in a continuum from benign to malignant tumours, suggesting the malignant transformation potential of endometriosis. Moreover, similar cancer driver gene mutations and altered molecular pathways have been observed in both endometriosis and EAOCs [17,18,19,20,21,22].

One of the survival pathways implicated in the development and progression of endometriosis and its associated EAOCs is the mechanistic (formerly the mammalian) target of rapamycin (mTOR) [23]. mTOR is a protein serine/threonine kinase which belongs to the phosphatidylinositol-3 kinase-related kinases (PIKKs) family [24] and plays a crucial role in maintaining cellular homeostasis by adjusting the balance between the anabolic and catabolic processes in response to environmental conditions [25]. Upstream regulators of mTOR include growth factors, nutrients, cellular energy, oxygen status, and genotoxic stresses [23]. The major anabolic downstream targets of mTOR are components involved in protein translation, angiogenesis, and lipid and protein biosynthesis. Autophagy and apoptosis are catabolic pathways negatively regulated by mTOR [26,27]. The aberrant activation of this pathway appears to favour carcinogenesis through the upregulation of protein translation, lipid biosynthesis, and angiogenesis, in addition to the inhibition of autophagy and apoptosis [26,27,28].

This review discusses the mTOR signalling pathway and its role in tumorigenesis, with an emphasis on endometriosis and EAOC. We then evaluate the existing literature on postmenopausal endometriosis and its malignant transformation potential. Finally, we conclude with the current updates on mTOR as a therapeutic target in EAOCs.

## 2. mTOR Signalling Pathway and Its Role in Tumorigenesis

### 2.1. mTOR Complexes

The mTOR is found in two spatially and functionally distinct multiprotein complexes, namely mTORC1 and mTORC2 [29,30].

mTORC1 consists of mTOR (the catalytic subunit), raptor (regulatory-associated protein of mTOR), PRAS40 (proline rich AKT substrate 40 kDa), Deptor, and mLST8 (mammalian lethal with sec-13), also known as s GbetaL [30]. The activation of mTORC1 requires interactions with several binding partners and translocation of mTORC1 within the cell. Growth factors and amino acids activate mTORC1 through two different types of small GTPases-Ras-homolog enriched in brain (Rheb) and the Rag GTPases [31]. Amino acids cause Rag GTPase to switch to active conformation. Active Rag GTPase interacts with the mTORC1 subunit raptor, translocating the complex from the cytoplasm to the lysosomal membrane where Rheb resides [31]. Rheb binds directly to mTOR, inducing conformational changes, suggesting an allosteric mechanism for activating TORC1 [32]. Growth factors induce mTORC1 activation via the PI3K–AKT signalling pathway and the regulation of a small GTPase protein Rheb via the tuberous sclerosis complex (TSC1/TSC2), a potent negative regulator of mTORC1 [33]. PRAS40 inhibits mTORC1 and is found bound to the substrate binding site of raptor. When PRAS40 is phosphorylated by AKT, it dissociates from the mTORC1 complex, revealing the substrate binding site, allowing the binding and activation of mTORC1 substrates like ribosomal protein S6 kinase 1 (S6K1) and 4E-BP1 [34]. mLST8 stabilizes the active site of mTOR, but its precise function has not been defined yet [35].

The mTORC2 complex is comprised of mTOR, mLST8, rictor (raptor-independent companion of mTOR), mSIN1 (mammalian stress-activated protein kinase interacting protein 1), Protor-1 (protein observed with rictor-1), and Deptor [36]. The mechanism of mTORC2 activation, as well as its downstream signalling pathway(s) and partner protein interactions are not fully elucidated. However, SIN1 appears to stabilize and tether rictor to the mTOR-mLST8 core and plays a significant role in mTORC2 activity [36,37,38,39]. SIN1 further uses mLST8 as a platform for positioning its substrate-recruiting CRIM (conserved region in the middle) domain. mLST8 ablation in mice experiment led to a complete loss of mTORC2 activity, indicating its importance as a core component in the mTORC2 complex [37,39].

Deptor (DEP-domain containing mTOR-interacting protein) bound to mTOR suppresses its kinase activity in both mTORC1 and mTORC2 complexes. Deptor is also a substrate of the activated mTORC1 complex, facilitating its degradation [40]. Mechanistically, activated mTORC1 phosphorylates Deptor, marking it for ubiquitination. The tagged protein is then shuttled to the proteasome, the cellular machinery responsible for degrading proteins [41]. The dual role of Deptor as both an inhibitor of mTOR, and a substrate of activated mTORC1, positions the protein as a central player in determining the activity status of the mTOR pathway. Interestingly, Broadway et al. suggested the potential role of Deptor as a prognostic biomarker, since its upregulation appears to be positively correlated to better overall survival in ovarian cancer patients [23].

### 2.2. mTOR Pathway: Upstream Regulators and Downstream Effectors

**Figure 1:**
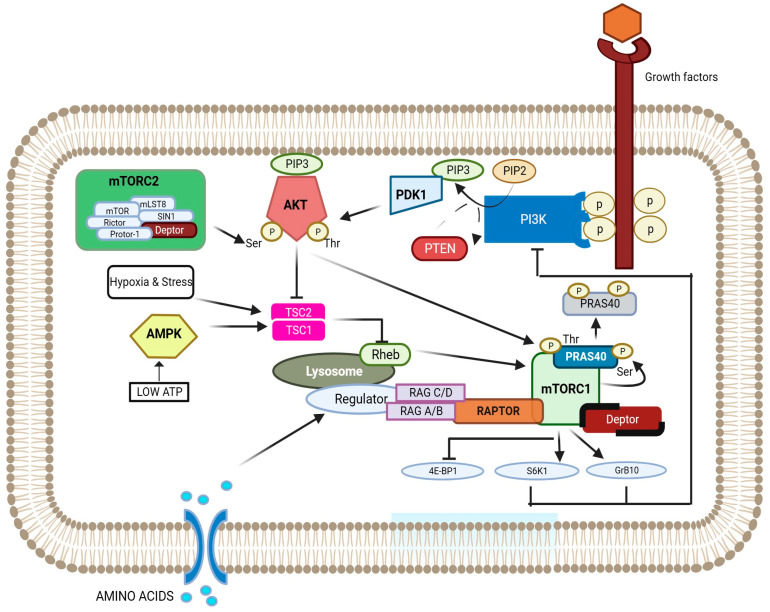
The binding of growth factors to receptor tyrosine kinase (RTK) results in the activation of phosphatidylinositol 3′-kinase (PI3K). Activated PI3K phosphorylates phosphatidylinositol-4,5-biphosphate (PIP2) to form phosphatidylinositol-3,4,5-triphosphate (PIP3). PIP3 binds to the pleckstrin homology domains of phosphoinositide-dependent kinase 1 (PDK1) and mediates the phosphorylation of AKT on Thr308. The phosphorylation of AKT-Ser473 is mediated by mTORC2. Activated AKT then promotes the phosphorylation of Thr246 on PRAS40. In addition, AKT inhibits the activity of the tuberous sclerosis complex (TSC1)/TSC2 complex, which results in increases in the levels of GTP-bound Rheb and the activation of mTORC1. Activated mTORC1 phosphorylates multiple protein substrates, including PRAS40, 4E-binding protein 1 (4E-BP1), ribosomal protein S6 kinase 1 (S6K1), and growth factor receptor bound 10 (Grb10). The phosphorylation of PRAS40 results in dissociation from the mTORC1 complex. Amino acids can stimulate mTORC1 complex as they cause Rag GTPase to switch to active conformation. Active Rag GTPase interacts with mTORC1 subunit raptor, translocating the complex from the cytoplasm to the lysosomal membrane where Rheb resides. Rheb directly binds and activates mTOR. In contrast to growth factors, cellular stresses, hypoxia, and energy deprivation promote the activity of the TSC1/TSC2 complex via AMP-activated protein kinase (AMPK) phosphorylations of TSC2, thus resulting in the inhibition of the mTORC1 pathway. The tumour suppressor protein phosphatase and tensin homolog (PTEN) reverses PIP3 to PIP2 and antagonizes the PI3K–AKT mTOR pathway. Image adapted from Wiza et al. [34].

One of the key upstream regulators of mTORC1 is the phosphatidylinositol 3-kinase (PI3K)–AKT pathway. Growth factor-activated receptor tyrosine kinase (RTK) promotes PI3K activation leading to the phosphorylation of phosphatidylinositol-4,5-phosphate (PIP2) to phosphatidylinositol-3,4,5-phosphate (PIP3), subsequently activating AKT. Active AKT promotes mTORC1 action in two ways: (1) reducing the interaction of proline-rich AKT substrate 40 kDa (PRAS40) with mTORC1 and (2) phosphorylating and inactivating the tuberous sclerosis complex (TSC1/TSC2) (also called hamartin and tuberin) [42,43]. Within the complex, TSC2 acts as a GTPase-activating protein (GAP) for the Rheb GTPase and is stabilized by TSC1. TSC2 inactivation by AKT-dependent phosphorylation destabilizes TSC2 and disrupts its interaction with TSC1, thus relieving its inhibitory constraint on Rheb [44]. The GTP-bound form of Rheb directly interacts with mTORC1 and stimulates its kinase activity [44]. The tumour suppressor protein phosphatase and tensin homolog (PTEN) reverses PIP3 to PIP2 and antagonizes the PI3K–AKT mTOR pathway [43]. Nutrients and cellular energy levels further regulate mTORC1 activity through different mechanisms and convergent pathways (Figure 1). Downstream effectors of mTORC1 include eukaryotic translation initiation factor 4E-binding protein (4EBP), p70 S6 kinase (S6K), and UNC-51-like kinase (ULK1) [45].

mTORC2 is less sensitive to nutrients and energy levels and more responsive to insulin and growth factors. The activation mechanism of mTORC2 is proposed to follow two steps. First, growth factors induce the initial partial activation of AKT on Thr308. This activation is sufficient to directly phosphorylate SIN1 within the mTORC2 complex, thereby enhancing its kinase activity. Subsequently, the increased kinase activity of mTORC2 facilitates the full activation of AKT by phosphorylating it at Ser473 [45,46]. The main substrates of mTORC2 are members of the AGC kinases, including AKT, (protein kinase C) PKC, and (serum- and glucocorticoid-inducible kinase 1) SGK-1 [47].

### 2.3. mTOR Pathway Role in Tumorigenesis

The mTOR pathway is a key player in the metabolic reprogramming of cancer cells. Both normal and cancer cells metabolize nutrients, mainly glucose, to produce energy in the form of ATP. There are two ways of producing energy: The first is through glycolysis, an anaerobic process which does not require oxygen [48]. The second is via respiration in the mitochondria, which requires oxygen and produces much more energy. In order to gain access to nutrients under a hypoxic tumour microenvironment, the pathway mediates a shift from oxidative phosphorylation in the mitochondria to glycolysis [49]. The total number of ATPs produced through glycolysis is far less than through oxidative phosphorylation. Notably, the activation of mTOR increases GLUT1 expression, a membrane protein that facilitates the transport of glucose into the cell, and HK2 (an enzyme involved in the nine-step glycolysis reaction), subsequently leading to an increase in the glycolysis rate [50]. Moreover, cancer cells increase their de novo production of lipids to generate ATPs. The de novo lipogenesis is mainly regulated at the transcriptional level by activating regulatory element-binding proteins (SREBPs). SREBPs are present as inactive precursors in the endoplasmic reticulum (ER), whilst upon activation, they translocate to the Golgi apparatus, where they undergo proteolytic cleavage processing, releasing mature, transcriptionally active SREBPs. The mature SREBPs translocate to the nucleus and bind to the promoter regions of target genes, involved in de novo lipid biosynthesis. The activation of SREBP1, a specific isoform of SREBPs, involves the ribosomal protein S6 kinase beta-1 (S6K1) activation [51,52].

A recognized downstream effector of the mTOR pathway is the eukaryotic initiation factor 4E binding protein-1 (4E-BP1). Upon phosphorylation, it dissociates from the mRNA cap-binding protein eukaryotic translation initiation factor 4E (eIF4E) and promotes protein synthesis required for cell growth [53]. mTOR phosphorylation of the ribosomal protein S6K1 stimulates protein translation, which is required for cell growth and G1/S cell cycle progression. Subsequently, the downregulation of the mTOR pathway, downregulates cyclin/CDK complexes, particularly cyclin D1 and CDK4, and blocks the cell cycle in the late G1/S phase [54,55].

Autophagy is defined as the intracellular lysosomal degradation and recycling of cell organelles and misfolded proteins. The function and activation of autophagy-related genes is tightly regulated by nutrient supply (via mTOR), energy availability (via AMP-activated protein kinase AMPK), and stress (via hypoxia-inducible factors HIFs) [28,56]. The regulatory mechanisms of autophagy significantly overlap with signalling pathways associated with tumorigenesis. Notably, tumour suppressor genes like PTEN, which inhibit mTOR signalling, act as facilitators of autophagic processes. Conversely, oncogenic entities, including PI3K, which amplify mTOR signalling, attenuate autophagic activity [57,58]. Under nutrient-rich conditions, mTORC1 inhibits autophagy through the regulation of a protein complex composed of unc-51-like kinase 1 (ULK1). Conversely, energy starvation activates the 5′-AMP-activated protein kinase (AMPK) pathway, which phosphorylates ULK1 and initiates autophagy [28].

Autophagy, in general, can serve as a cell survival or cell death mechanism, and its role in cancer seems ambivalent [56,59]. Both induction and inhibition can be pro- or anti-tumorigenic. When cancer is growing, hypoxia and starvation upregulate autophagy to maintain the nutrient abundance required for cancer progression. Furthermore, it enables tumour cells to endure the chemotherapy-induced oxidative stress and enter dormancy, resulting in chemotherapy resistance and cancer recurrence [60].

## 3. The role of mTOR Pathway in Endometriosis and Endometriosis-Associated Ovarian Cancers (EAOCs)

### 3.1. Epidemiology and Pathogenesis of Endometriosis

Despite the clinical acceptance of Sampson’s retrograde menstruation theory, several other hypotheses regarding the pathogenesis of endometriosis have been suggested [61]. The coelomic metaplasia theory proposed a metaplastic transition of mesothelial cells into ectopic endometrium [62]. Some authors theorized a differentiation process of mesenchymal cells, activated by chemicals released from the degenerating endometrium which reaches the abdominal cavity [63]. The stem cell theory also assumes a differentiation process of pluripotent stem cells, which, under certain circumstances, gives rise to endometrial cells [64].

Endometrial tissue outside the uterine cavity is the hallmark of endometriosis. When the endometrial tissue lies within the myometrium, it is called adenomyosis. Adenomyosis is a benign gynaecological disease often associated with pelvic pain and infertility [65]. Despite the clinical differences between endometriosis and adenomyosis, the two conditions may actually represent two phenotypes of a single disease [65].

Diagnosis of endometriosis is currently clinical and relies on imaging and visualizing endometriotic lesions during laparoscopy. Three different forms of endometriosis exist, namely peritoneal, ovarian, and deeply infiltrating lesions (DIE). Peritoneal and ovarian implants can be white, red, or black lesions. The red lesions are highly vascular and represent early disease while the white lesions are old fibrotic scars. The black lesions are essentially enclosed implants with intraluminal debris of tissue breakdown [9,62]. Several classifications and staging reporting systems have been developed [66]. The most clinically accepted is the Revised American Fertility Society (rAFS)/Revised American Society for Reproductive Medicine (rASRM) classification [67]. The rASRM staging system categorizes endometriosis into four stages based on the extent and severity of the disease. These stages range from minimal (Stage I) to mild (Stage II), moderate (Stage III), and severe (Stage IV). This classification considers factors such as the location and depth of endometrial implants, the presence of adhesions, and the involvement of other pelvic structures [67]. The European Society of Human Reproduction and Embryology (ESHRE) recommends histological confirmation of endometriosis as a standard part of the diagnostic workup, with a positive identification of endometrial-like glands and/or stroma within the biopsied samples [68,69].

Although normal and ectopic endometrium are histologically similar, endometriotic lesions show a dysregulated response to ovarian steroids [70,71]. Oestrogen (E2) and progesterone are the master regulators of endometrial tissue. Each hormone regulates the expression of hundreds of genes during various phases of the menstrual cycle. In eutopic endometrium, E2 induces epithelial proliferation during the proliferative phase of the cycle, and then progesterone (P4) inhibits E2-induced proliferation during the secretory phase. E2-induced protein and DNA synthesis in endometrial tissue is mediated via the mTOR pathway [72]. Choi et al. demonstrated a higher expression level of phosphorylated p70S6K during the early proliferative phase compared to the secretory phase in normal endometrial cells. This higher expression level was also seen in cultured endometrial cells with oestrogen alone compared to those treated with oestrogen and progesterone. However, the expression levels remained unchanged in cultured ectopic endometrium with oestrogen and progesterone [73]. Endometriotic lesions, in general, are distinctive in two ways: (i) high levels of local oestrogen production and (ii) progesterone resistance. The high level of local oestrogen is attributed to the presence of a full complement of enzymes that convert androgens into oestrogens, adding to the proliferative effect of the circulating oestrogen on the endometriotic tissues [74]. Progesterone and its receptor isoforms, PR-A and PR-B, also have established roles in endometriosis. Several causes of progesterone resistance have been postulated, including congenital “preconditioning”, genetics, and environmental causes. Progesterone resistance results into a pro-inflammatory phenotype. Subsequently, repetitive chronic inflammation increases progesterone resistance. Of note, the eutopic endometrium in women with endometriosis shows a degree of progesterone resistance [9,70].

### 3.2. mTOR Pathway Aberrations in Endometriosis and Endometriosis-Associated Ovarian Cancers (EAOCs)

Over the past decade, cumulative evidence has implicated certain intracellular signalling pathways dysregulation in the molecular pathogenesis of endometriosis [71]. The mTOR pathway has been extensively studied as a potential pathway underpinning the initiation and development of endometriosis [75]. Eutopic endometrium appears to play a role in the development of endometriosis [76]. In an early study by Cinar et al., it was shown that AKT activity was elevated in both the eutopic and ectopic endometrium of women with endometriosis, with endometriotic glandular cells demonstrating significantly higher levels of AKT activity when compared to the normal endometrium [77]. A recent transcriptome meta-analysis comparing the eutopic endometrium of women with stage III–IV endometriosis to normal endometrium from healthy counterparts demonstrated the enrichment of the PI3K, AKT, mTOR, and TGF signalling pathways [78].

Indeed, AKT hyperactivity plays a primary role in the development of endometriosis. Kim et al. showed that uterine cells lacking PTEN developed more endometriotic lesions compared to those with intact PTEN in vivo. Furthermore, a significant reduction in endometriotic lesion numbers was noted when (PRcre/+Ptenf/+) ovariectomized mice with surgically induced endometriosis were treated with the AKT inhibitor MK-2206 [79]. PTEN expression in normal endometrium is subjected to progesterone control. As progesterone secretion increases towards the second half of the menstrual cycle (the secretory phase), PTEN expression increases. [80]. Autophagy homeostasis is detrimental to endometriotic cells; whilst moderate autophagic response acts as a housekeeping and survival mechanism, the extensive activation of autophagy results in autophagic cell death [81]. Endometriotic cells are progesterone-resistant and hence have constantly suppressed levels of PTEN, irrespective of the menstrual cycle phase [80]. Choi et al. demonstrated an inverse correlation between p70S6K (downstream effector of the PI3K/AKT/mTOR pathway) and LC3-II (autophagic cell markers), indicating the negative impact of the mTOR pathway activation on autophagy [73]. A constant expression of p70S6K and LC3-II in the endometriotic cells, irrespective of the menstrual cycle phase, was also observed in the same study [73,82].

Endometriosis is primarily an oestrogen-dependent condition. At the molecular level, oestrogen biological effects are mediated via two types of receptors (ERs): nuclear (ERα and ERβ) and the membrane receptor G protein-coupled oestrogen receptor 1 (also known as GPER or GPR30) [83,84]. The classic oestrogen signalling pathway is mediated via ERα and ERβ receptors, which, upon activation, are translocated to the nucleus to modulate the transcription of target genes. ERβ receptors are overexpressed in endometriotic tissues compared to normal endometrium, whilst ERα has significantly lower levels of expression [74]. ERβ directly induces Ras-like oestrogen-regulated growth inhibitor (RERG) gene expression, consequently enhancing the proliferative activity of endometriosis. ERβ also suppresses ERα gene expression, inhibiting its mediated progesterone receptor (PR) expression. The full spectrum of ERβ functions is probably more intricate, considering the notably heightened levels of ERβ found in both nuclear and cytoplasmic locations within endometriotic tissues [85]. Beyond the genomic slow mechanism, oestrogen also triggers a non-genomic rapid effect through its membrane receptor (GBER). This receptor can induce the transactivation of the epidermal growth factor receptor (EGFR), subsequently activating various downstream effectors, including PI3K [86]. Moreover, the expression of GBER has been observed to be influenced by stress hormones and inflammation, which are hallmark features of the endometriosis microenvironment [87]. The GPER agonist known as G-1 has been shown to inhibit proliferation and promote apoptosis in endometrial stromal cells, indicating its potential use in the treatment of endometriosis [88].

As aforementioned, endometriosis is not a pre-cancerous condition and is better described as a benign disease with malignant potential, with a malignant transformation of endometriosis occurring in about 1–2% of the patients [6,89,90]. Ultimately, those with endometriosis face a heightened risk of developing ovarian cancer, with odds ratios ranging from 1.3 to 1.9 [91,92]. This means that the overall risk of developing ovarian cancer in those with endometriosis is 1.8%, compared to 1.31% in the general population [93].

Ovarian cancers developing in endometriosis are far more likely to be clear-cell or endometrioid adenocarcinoma than any other histological subtypes[6,94]. Criteria to define tumours as EAOCs were first described by Samson in 1925 and later refined by Scot, stating that benign endometriosis should be contiguous to the cancer tissue with a histologically proven transition to cancer [95]. Since then, several retrospective and epidemiological studies have reported the concurrent presence of endometriosis adjacent to the malignant tumour in a continuum from benign to malignant in clear-cell and endometrioid adenocarcinomas [96,97]. Although clear-cell and endometrioid subtypes are often grouped as EAOCs, a histogenesis dichotomy has been suggested. It has been proposed that the clear-cell subtype is more likely to arise from endometriosis as its precursor lesion, while the endometrioid subtype may result from Müllerian metaplasia. However, the molecular changes underlying the development of the two subtypes have shown commonalities, particularly regarding mTOR dysregulation. Further investigation into molecular aberrations in these two subtypes is warranted before affirming such a dichotomy [98].

The concept of “atypical endometriosis” evolved over time, describing a non-invasive intermediate stage characterized by cytological atypia and architectural disorganization [99]. The presence of atypical endometriosis adjacent to the tumour mass in continuation with benign endometrium led to the belief that it may represent early stages of malignant transformation [6,91,100,101]. Gabriele et al. suggested a clinical treatment algorithm based on the presence or absence of atypical endometriosis [102]. However, the presence of cancer driver genes’ mutations in seemingly normal endometriotic tissue adjacent to the tumour without histological atypia underscores the urgency of comprehending the molecular pathways driving the tumorigenesis of endometriosis (Table 1) [103,104,105].

Endometriotic lesions also harbour cancer driver mutations such as PIK3CA, PTEN, ARID1A, KRAS, PPP2R1A, and *β*-catenin (CTNNB1) (Table 1). These mutations are implicated in the malignant transformation potential of endometriosis in a complex interplay with the tumour microenvironment [18]. The PIK3CA gene encodes the p110α catalytic subunit of PI3K. Somatic alterations of PIK3CA through mutations or gene amplification result in the aberrant activation of the PI3K–AKT–mTOR signalling pathway [106]. PIK3CA mutants in ovarian cancers are seen at hotspot sites in exons 9 and 20 [106]. Yamamoto et al. reported identical PIK3CA mutations in the synchronous endometriotic epithelium in patients with ovarian clear cell carcinoma. These mutations were observed in both atypical and non-atypical endometriotic tissues, suggesting PIK3CA mutations as very early events in ovarian clear-cell carcinoma development [107,108]. Similar findings were demonstrated by Matsumoto et al. for both ovarian clear-cell and endometrioid subtypes [103]. Recently, a number of studies have shown PIK3CA mutations in eutopic endometrial glands in women with and without EAOCs and endometriosis, suggesting that these mutations may confer a survival advantage, allowing for a clonal expansion of these cells at the ectopic sites and are not sole direct driver of tumorigenesis. The low frequency of gene mutation in eutopic endometrium and in the benign endometriotic epithelium unrelated to ovarian clear-cell adenocarcinoma (OCCC) may only reflect sporadic PIK3CA mutations in endometriotic and eutopic endometrial glands [109,110,111].

Somatic PTEN mutations have been observed in the endometrium of women with endometriosis as well as in endometriosis and endometriosis-associated ovarian cancers [109,110,112], indicating that the inactivation of the PTEN tumour suppressor gene is an early event in the development of ovarian endometrioid and clear-cell adenocarcinoma [113,114].

ARID1A (AT-rich interaction domain 1A) is the largest subunit of the SWI/SNF (switch/sucrose non-fermentable) complex and plays an important role in chromatin remodelling and tumour suppression [115]. The mutation status of the AIRDA1A gene determines the protein expression level and progression to cancer [116,117,118]. The two alleles of the gene need to acquire loss-of-function mutations for a complete loss of protein expression and progression to cancer [119]. Therefore, ARID1A mutations demonstrated in endometriotic lesions adjacent to ovarian cancer and at distal sites vary in the resulting AIRD1A protein level. These findings support Knudson’s two-hit hypothesis, which proposes that the inactivation of both alleles of tumour suppressor genes is essential to cause a phenotypic change, leading to carcinogenesis [120]. ARID1A mutations are seen in ~50% of ovarian clear-cell cancers and ~30% of ovarian endometrioid carcinomas [116,121,122]. ARID1A inactivation alone is not enough to initiate carcinogenesis; additional concurrent genetic alterations, such as a mutation in PIK3CA or a PTEN deletion, are required to drive tumorigenesis into clear-cell or endometrioid carcinomas [107,122,123]. A concurrent loss of AIRD1A expression in both OCCCs and adjacent endometriotic epithelium were observed with a preservation of AIRD1A expression in distant endometriosis, implying its role in the malignant transformation of endometriosis. Interestingly, these mutations were observed in the adjacent histologically normal endometriotic tissues that did not necessarily show atypical features [104]. The ARID1A loss of expression and PIK3CA mutations coexisted frequently in a study by Yammato et al. and were not mutually exclusive [104]. In a conditional knockout mouse model, the double deletion of ARID1A and PTEN in the mouse ovarian surface epithelium led to the formation of ovarian endometrioid or undifferentiated carcinoma [124]. Collectively, these findings suggest that despite PTEN and PI3KCA mutations being early neoplastic transformations of endometriosis, it is not until multiple loss-of-function mutations of ARID1A, or a combination of oncogene and gene suppressor mutation co-exist, that complete cellular transition to malignancy takes place [109,125,126]. The direct or indirect inhibition of the PI3K/AKT/mTOR pathway leads to the synthetic lethality of ARID1A-deficient tumour cell clones [127,128].

Suda et al. demonstrated a recurrent occurrence of KRAS and PIK3CA mutations in both the endometriotic and normal endometrial epithelium. However, the frequency of these mutations in the endometriotic epithelium was much higher. The author proposed that endometrial tissues with KRAS mutations undergo retrograde transport to the ovarian surface. These specific KRAS mutations confer selective advantages, promoting endometriosis development and facilitating clonal expansion throughout the endometriotic lesion [129].

Cancer-associated mutations have been observed in deep infiltrating endometriosis (DIE), a type of endometriosis that rarely transforms into cancer, with a mere number of cases reported across the literature. This underscores the significant interplay among factors such as inflammatory reactions, hormone imbalances, and reactive oxygen species (ROS) in the pathogenesis of EAOCs, raising the question of whether somatic mutations in “cancer-associated genes” are sufficient for a malignant transformation [110,130]. Collectively, the activation of the mTOR pathway suppresses cell death. The ongoing insult in the endometriosis microenvironment through haem oxidative stress and hypoxia leads to the accumulation of genetic and epigenetic aberrations which eventually leads to cancer development [131,132,133,134].

The hypoxia-regulated gene network includes angiogenesis, inflammation, steroidogenesis, and metabolic switch. The activation of the hypoxia-inducible factor-1 alpha (HIF-1α) transcription factor is the most recognized pathway adopted by hypoxic cells in this harsh microenvironment [135]. Activated HIF-1α plays a crucial role in the adaptive responses of the cells to changes in oxygen through the transcriptional activation of over 100 downstream genes, which regulate vital biological processes required for survival and progression. The upregulation of ERβ and the downregulation of ERα observed in endometriosis is regulated at the transcriptional level by HIF-1α [136]. Hypoxia-induced angiogenesis in endometriosis is multifaceted, with HIF-1α expression postulated to increase a number of angiogenic factors, including vascular endothelial growth factor A (VEGF-A), leptin, IL-8, cysteine-rich protein 61 gene (CYR61), osteopontin (OPN), and fibroblast growth factor 9 (FGF9) [137,138,139]. The PI3K/AKT/mTOR signalling pathway contributes to the development of cancers by regulating HIF-1a activation; blocking the PI3K/AKT pathway inhibits HIF-1a expression and promotes its degradation [139,140].

Epigenetic regulation further modulates mTOR activity in EAOCs. For example, the IncRNA HCG11, a non-coding RNA, appears to suppress AKT/mTOR-mediated cell growth in ovarian cancer via the upregulation of PTEN activity, suggestive of an epigenetic modulation of mTOR [141]. Similarly, MFG-E8 siRNA, another non-coding mRNA, has been implicated in the AKT/mTOR/S6K signalling pathway in ovarian cancer cells [142]. Moreover, neighbouring cells in the growing cancer mass crosstalk through exosomes (i.e., extracellular double-membrane vesicles carrying regulating non-coding RNA which is introduced between cells), thus further regulating cellular activities, including mTOR regulation [143,144].

**Table 1:** Cancer driver mutations in endometriosis.

Study	Year	Endometriosis Location	Patients’ Age (Median/Range)/Menopausal Status	Endometriosis Stages	Endometriosis Morphology	Sample Size	Gene Mutation	Mutation Frequency	
Sato N et al. [113]	2000	OE ^1^	Not mentioned	All stages	Solitary endometrial cysts of the ovary	34	PTEN ^3^	OE	20%
Govatati S et al. [114]	2013	OE	Premenopausal	III/IV	Benign endometriosis	32	PTEN	OE/PE	53.10%
		PE ^2^							
Zou Y et al. [145]	2018	OE	32 (21–50)	not mentioned	Benign ovarian endometrial cysts	101	KRAS ^4^, PPP2R1A ^5^, ARID1A ^6^ co-occurrence of KRAS and AIRD1A in one patient	OE	4%
Xiao W et al. [146]	2012	AE ^7^	Not mentioned	all stages	Benign ovarian endometrial cysts. Histologically atypical endometriosis adjacent to OCCC ^8^	13 AE	ARID1A (loss of function mutation)	AE	38.5%
		OE				36 OE		OE	19.4%
Samartzis EP et al. [117]	2014	DIE ^9^	35 (25–42)	All stages	Benign ectopic typical endometrial tissue	22 DIE	ARID1A (loss of function mutation)	DIE	5%
		OE	35 (19–48)			20 OE		OE	15%
		PE	31 (25–38)			16 PE		PE	0%
Borrelli GM et al. [147]	2016	DIE	All premenopausal; only one ovarian endometrioma in postmenopausal woman	All stages	Benign ectopic typical endometrial tissue	25 DIE	ARID1A (loss of function mutation)	DIE	36%
		OE				20 OE		OE	30%
Chene G et al. [123]	2015	OE	Not mentioned	--------	Contiguous typical endometriosis to OCCC	66 OE	ARID1A (loss of function mutation)	OE	8%
		CE ^10^				18 CE		CE	44%
Anglesio MS et al. [110]	2017	DIE	37 (23–51)	--------	Benign deep infiltrating endometriosis	39	Multiple somatic cancer driver mutations including ARID1A, PIK3CA ^11^, KRAS, and PPP2R1A	DIE	26%
Suda K et al. [129]	2018	OE	Pre- and postmenopausal	Not mentioned	Discovery cohort of 13 ovarian endometriomas; validation cohort of 94 ovarian endometriomas, all benign	107	Recurrent mutations in KRAS, PIK3CA, FBXW7 ^12^ PPP2R1A, PIK3R1 ^13^	KRAS and PIK3CA most common recurrent mutations.	
Yamamoto S et al. [104]	2011	CE	----------------		Endometriosis adjacent to ARID1A deficient clear-cell carcinoma—typical and atypical endometriosis was included	23	AIRD1A	AE	100%
								TE ^14^	86%
Matsumoto T et al. [103]	2015	CE	54.1 (22–8)	--------	Endometriosis contiguous to ovarian endometrioid (OEAC) ^15^ and clear-cell carcinoma (OCCC)	49	*β-*catenin (CTNNB1) ^16^ PIK3CA	*β*-catenin (CTNNB1) mutations in the OEAC contiguous endometriosis	
								TE	52.4%
								AE	73.3%
								OCCC contiguous endometriosis 0%	
								PIK3CA mutations in OEAC contiguous endometriosis	
								TE	25%
								AE	40%
								PIK3CA mutations in OCCC contiguous endometriosis	
								TE	14.3%
								AE	75%
Yamamoto S et al. [108]	2011	CE	50.7 (41–58)	-------	Endometriosis adjacent to PIK3CA deficient OCCC- typical and atypical endometriosis was included	10	PIK3CA	TE	75%
								AE	88%

^1^ OE = ovarian endometriosis, ^2^ PE = peritoneal endometriosis, ^3^ PTEN = phosphatase and tensin homolog, ^4^ KRAS = Kirsten Rat Sarcoma Viral Oncogene Homolog. ^5^ PPP2R1A = Protein Phosphatase 2 Regulatory Subunit Aalpha, ^6^ ARID1A = AT-rich interaction domain 1A, ^7^ AE = atypical endometriosis, ^8^ OCCC = ovarian clear-cell adenocarcinoma, ^9^ DIE = deep infiltrating endometriosis, ^10^ CE = contiguous endometriosis, ^11^ PIK3CA = Phosphatidylinositol-4,5-Bisphosphate 3-Kinase Catalytic Subunit Alpha,^12^ FBXW7 = F-Box and WD Repeat Domain Containing 7, ^13^ PIK3R1 = Phosphoinositide-3-Kinase Regulatory Subunit 1, ^14^ TE = typical endometriosis, ^15^ OEAC = ovarian endometrioid adenocarcinoma, ^16^ *β-*catenin (CTNNB1) = Catenin Beta-1.

## 4. Postmenopausal Endometriosis and the Risk of Malignant Transformation

As mentioned previously, endometriosis predominantly affects women in their reproductive years, yet has been observed in 2–4% of postmenopausal women [12]. Since the disease is oestrogen-dependent, the conventional understanding would anticipate a regression of endometriosis with the decline in oestrogen levels after menopause. However, oestrogen and progesterone receptors appear to remain equally expressed in pre- and postmenopausal women, indicating a potential for reactivation of the disease in the presence of appropriate stimulation [15]. The “oestrogen threshold” theory suggests that a certain level of oestrogen is required to re-activate the existing endometriosis [148].

HRT and obesity are exogenous and endogenous oestrogen sources, respectively. These can potentially increase the risk of endometriosis recurrence and cancer development, particularly clear-cell and endometrioid carcinoma [15]. Long-term use of oestrogen-only HRT, premenopausal hysterectomy, and previous history of endometriosis are all risk factors for a neoplastic transformation of endometriosis [149,150]. Tamoxifen, a selective oestrogen receptor modulator (SERM) with an agonist effect on endometrial tissue, can potentially have a similar effect on endometriosis [149].

Furthermore, endometriotic lesions express a full complement of enzymes required for oestrogen synthesis, suggesting local oestrogen production within the lesion’s microenvironment [151,152,153,154,155]. Aromatase and steroidogenic acute regulatory protein (StAR) are key players in local oestrogen production. High expression levels and enzyme activity have been demonstrated in cultured stromal cells from endometriotic lesions [154,155]. Whether endometriosis can develop de novo in this age group is unclear as there are a number of premenopausal women with asymptomatic endometriosis [14]. A case series of seven women who developed endometriosis ten years after the menopause supported the genetic and epigenetic theory of endometriosis development, i.e., endometriosis developed as a result of a cumulative series of genetic or epigenetic incidents [156].

Furthermore, menopause marks a significant shift in endocrine and immunological equilibrium, potentially influencing the relevance of genetic factors. Watrowski et al. demonstrated a significant association between single-nucleotide polymorphisms’ (SNPs) genetic variation of interleukin-8 (IL-8), a pro-inflammatory and pro-angiogenic chemokine often altered in endometriosis and cancers and implicated in the activation of the PI3K/Akt pathway, and EAOC [157].

Symptoms of postmenopausal endometriosis are non-specific and may include abdominal pain, vaginal bleeding, gastrointestinal symptoms, rectal bleeding, and ovarian masses [158]. However, this age group should be treated with high suspicion of malignant changes. The first-line treatment for women with postmenopausal endometriosis is surgical. Medical treatment may include aromatase inhibitors and, hypothetically, progesterone, although there are no reported cases on the use of progesterone in postmenopausal women [159,160]. Postmenopausal endometriosis is less active and less extensive than premenopausal endometriosis [158]. The disease is likely to present as ovarian endometriomas or deep infiltrating endometriosis, and the pattern of superficial peritoneal endometriosis is rarely seen in this age group [161].

A malignant transformation of endometriosis occurs in 1–2% of all cases; this risk increases with age [160,162]. Most EAOCs occur in perimenopausal women; a 2021 systematic review looking at postmenopausal women with a malignant transformation of endometriosis reported a mean age of 55.8 ± 8.6 years with almost two-thirds of these patients reporting a previous personal history of endometriosis [149]. A malignant transformation of endometriosis, although associated with the ovaries in 80% of the cases, has also been reported in extraovarian locations such as the abdominal wall, rectovaginal septum, and intestine [163]. Interestingly, malignant transformations occurring in the ovaries are typically treated with chemotherapy, in line with the treatment protocols for ovarian cancer. Conversely, when such transformations occur extra-gonadally, such as in the rectum or recto-sigmoid, then the treatment becomes surgical resection and radiotherapy [164,165]. Adding to the controversy, the management of malignant transformations of abdominal wall endometriosis varies; at times, they are approached akin to advanced endometrial cancer with a combination of radiotherapy and chemotherapy, while in other instances, they are treated similar to ovarian cancer with platinum-based chemotherapy alone [166].

Oestrogen mediates a plethora of molecular changes, including transcription and translation, via the mTOR pathway in the endometriotic cells [167]. Active phosphorylated mTOR expression was found to be 3.5-fold higher in postmenopausal endometriosis compared to premenopausal counterparts. Furthermore, active mTOR was not significantly different in ovarian carcinoma compared to postmenopausal endometriosis [168]. However, these cells remained morphologically benign with no evidence of atypia or malignant transformation [168]. There is no doubt that time is an essential factor in cancer development, hence postmenopausal endometriosis has a greater predisposition to malignant transformation [161].

However, a systematic review investigating risk factors for developing EAOC among women with endometriosis highlighted a subset of women at increased risk of malignant transformation, perhaps irrespective of menopausal status. This subset of women included an older age at endometriosis diagnosis (≥45 years, pre- or postmenopausal), nulliparity, hyperestrogenism (endogenous or exogenous), the premenopausal status at the endometriosis diagnosis, solid compartments, as well as a larger size of endometrioma (≥9 cm in diameter at endometriosis diagnosis); all were associated with an increased risk of EAOC [169].

## 5. Targeting mTOR Pathway in Endometriosis and EAOC Treatment

The heightened activity of the PI3K/mTOR pathway, observed in both endometriosis and EAOCs, underscores its potential as a promising therapeutic target [170,171]. In fact, the mechanistic target of rapamycin acquired its name from the first described mTOR inhibitor, “rapamycin”.

Rapamycin was first described in 1975 as an antifungal antibiotic produced by a bacterial strain isolated from the soil of Rapa Nui (Easter Island) [172]. By 1990, the drug’s immunosuppressive and anti-tumoral properties gained recognition [173,174]. However, rapamycin’s molecular target remained unclear until the mid-1990s, when mTOR became an active area of research discoveries [175,176]. Rapamycin targets this pathway predominantly through the inhibition of mTORC1 with very weak and time-dependent activity on mTORC2 [177]. Subsequently, rapalogues were developed as semi-synthetic analogues of rapamycin. These also target mTORC1 by allosteric inhibition, forming a complex with cytosolic FK506-binding protein [178].

However, a serious drawback of the first-generation rapalogues was the compensatory activation of upstream pathways with no or partial block of mTORC2, eventually deregulating the entire mTOR network and compromising the inhibitory activity [179]. Hence, new generations of mTOR inhibitors with dual mTORC1 and mTORC2 (RapaLink 1), dual PI3K-mTOR inhibitors, PI3K inhibitors, and AKT inhibitors were developed [180,181,182].

The current European Society of Human Reproduction and Embryology (ESHRE) guidelines outlining the management of endometriosis advocates for either the surgical removal of endometriotic lesions or the implementation of hormonal and symptomatic treatments [68]. These two approaches lack long-term control and endometriosis often reappears. Dienogest (an orally active synthetic progestogen commonly used in the treatment of endometriosis) acts by inhibiting the PI3K–AKT and MEK1/2–ERK1/2 pathways in the endometriotic cells [183]. Ren et al. demonstrated a significant decrease in the volume of endometriotic lesions in rapamycin-treated mice [184]. Similarly, Kacan et al., showed promising results with everolimus (Afinitor^®^, Novartis, NJ, USA), a first-generation rapalogue [185]. MK2206, an AKT inhibitor, has also shown promising preclinical results in endometriosis [186]. However, to translate these findings into practical long-term endometriosis treatment, significant enhancements in clinical efficacy and a thorough evaluation of the adverse effect profile are imperative [187].

The gold-standard treatment for epithelial ovarian cancer is a combination of taxane- and platinum-based chemotherapeutics, irrespective of the clinical subtype [188]. The response rate of the standard chemotherapy in advanced ovarian clear-cell cancer (OCCC) is low, making it, except for early-stage disease, the poorest stage-adjusted prognosis when compared to other ovarian cancer subtypes [188,189]. To overcome standard treatment failure, alternative or adjunct therapeutics are needed. The PI3K/AKT/mTOR pathway is an appealing therapeutic target, given the high frequency of mutations in its regulatory proteins seen in EAOCs [190].

A number of in vivo and in vitro preclinical studies have investigated mTOR inhibitors for ovarian cancer treatment either alone or in combination with other cytotoxic drugs. Shi et al. demonstrated that rapamycin could effectively enhance cisplatin-induced apoptosis in platinum-resistant SKOV3 ovarian cancer cells in vitro [191]. In another study, everolimus inhibited the mTOR signalling pathway in ovarian cancer cells exhibiting elevated AKT/mTOR expression. In the same study, the authors reported enhanced cisplatin-induced apoptosis in SKOV3 and OVCAR10 cells treated with everolimus and the inhibition of tumour growth and angiogenesis in mouse SKOV3 xenograft models [192]. The dual mTORC1/mTORC2 inhibitor vistusertib (AZD2014), in combination with paclitaxel, reduced the tumour growth and increased apoptosis in the resistant xenograft model [193]. The dual PI3K/mTOR inhibitor GSK458 demonstrated a potent inhibition of proliferation and cell migration in combination with paclitaxel in vitro and reduced tumour growth in SKOV3 xenograft and PDCX models in vivo [194]. However, to date, no PI3K/AKT/mTOR pathway inhibitor has been approved by the U.S. Food and Drug Administration (FDA) for the treatment of EOCs.

A meta-analysis assessing the effectiveness of monotherapy with PI3K/AKT/mTOR pathway inhibitors in ovarian cancer reported an overall low response rate (ORR) of 3% in ovarian cancer patients. A sub-analysis by the inhibitor group showed that PK3I inhibitors were associated with the highest pooled clinical benefit rate (CBR), whilst mTOR inhibitors were associated with the best ORR; however, there was no statistically significant difference between the groups [195]. Of note, dual PI3K/mTOR inhibitors have struggled to advance beyond phase I trials in numerous cancers, largely due to concerns related to compromised safety and the occurrence of frequent adverse events [182].

Alpelisib is a small PI3K inhibitor that selectively inhibits p110 and has been FDA-approved for patients with hormone receptor-positive (HR+) and human epidermal growth factor receptor 2-negative (HER2-) PIK3CA-mutant breast cancer. A phase III randomized study of alpelisib in combination with olaparib in patients with no germline BRCA mutations, platinum resistance, and high-grade serous ovarian cancer is currently recruiting [196].

Another phase II trial (DICE trial) has been initiated, investigating the addition of sapanisertib (TAK-228; an oral dual mTORC1/mTORC2 inhibitor) to paclitaxel in the treatment of advanced/recurrent epithelial ovarian, fallopian tube, or primary peritoneal cancer (clear-cell, endometrioid, and high-grade serous type, and carcinosarcoma) [197]. Table 2 presents an up-to-date summary of currently recruiting clinical trials involving PI3K–AKT–mTOR pathway inhibitors in ovarian cancer. The summarized data were obtained from clinicaltrial.gov and cover the period up to the end of February 2024, offering a comprehensive snapshot of the latest trials in this field.

Of note, patient selection based on current PI3K/AKT/mTOR biomarkers revealed a trend towards an improved clinical benefit rate in the meta-analysis investigating the effectiveness of monotherapy with PI3K/AKT/mTOR pathway inhibitors in ovarian cancer. However, this trend did not reach statistical significance for any such biomarker [195]. Commonly used predictive biomarkers in clinical trials to stratify patients for treatment include PIK3CA, PIK3R1, AKT2 gene mutations, and PTEN protein expression [195]. Significant advances are essential to fast-track new pathway inhibitors to clinical practice, including the characterization of new potential predictive biomarkers in the pathway and exploring different drug combinations.

## 6. Conclusions

The pathogenesis of endometriosis is complex, involving the activation of the mTOR pathway orchestrated by genetic and epigenetic mutations, which are ultimately implicated in its potential for malignant transformation. Postmenopausal endometriosis is particularly important, given that the peak age for cancer development is around menopause. The road from benign endometriosis to EAOCs is complex, yet most of the implicated cancer driver genes are upstream regulators of the mTOR pathway. Hypoxia, inflammation, and the immune microenvironment further regulate this pathway and may be essential for endometriosis-related cancer transformation, potentially positioning the mTOR pathway at the centre of multiple molecular pathways leading to cancer development. This may provide an opportunity to identify a precursor lesion to be targeted as a preventative or therapeutic strategy. Therapeutic targeting of the mTOR pathway may represent the future in preventing ovarian cancer and may offer potential predictive and prognostic biomarkers in EAOCs.

## 7. Future Directions

Ovarian cancer currently encompasses a heterogeneous group of subtypes that differ in their precursor lesions, prognoses, and biological behaviour [6,7,8]. Recent advances in the molecular classification of endometrial cancer indicate that future research in gynaecological cancers will increasingly focus on the molecular classification of cancer subtypes. While histological classification will continue to play a role, it is likely that molecular classification will eventually take precedence [198]. Ovarian cancer, however, remains under-investigated despite evolving in this regard, highlighting the urgent need for further molecular subclassification. This is crucial not only for identifying prognostic indicators but also for streamlining treatment pathways in the era of personalized medicine [5].

We hypothesize that the mTOR pathway plays a significant role in the development and progression of endometriosis-associated ovarian cancer (EAOC), and that it is dysregulated in this cohort of patients, potentially driving the transformation process. Investigating this pathway in EAOC patients could significantly impact treatment strategies, which currently rely heavily on surgery and platinum-based chemotherapy—often rendered ineffective due to resistance [167].

Furthermore, with the recent development of non-invasive endometriosis testing using salivary miRNA signature, there is a potential to evaluate this signature in EAOC. If proven to be present, such markers could serve as an effective tool for pre-cancer screening in this patient population. This approach could revolutionize the early detection and treatment landscape for ovarian cancer, aligning with the goals of personalized medicine [199].

Although numerous clinical and preclinical trials have evaluated mTOR inhibitors in ovarian cancer, several challenges have hindered their progression into clinical practice. These challenges include bypass activation pathways, off-target toxicities, and the lack of predictive biomarkers for patient selection and response to treatment [200]. A further evaluation of mTOR pathway inhibitors in both endometriosis and EAOC is necessary [167].

## Figures and Tables

**Table 2:** Currently recruiting clinical trials with PI3k–AKT–mTOR inhibitors for ovarian cancer treatment registered on ClinicalTrials.gov up to the end of February 2024.

Trial Title	Drug	Target	Trial	Patient Group	Study Design	Primary Outcome	Secondary Outcomes	First Posted	Clinical Trial ID
**A Study to Evaluate the Efficacy and Safety of CYH33 in Patients with Recurrent/Persistent Ovary Clear Cell Carcinoma**	CYH33	PI3K ^1^	Phase II study, single arm	Recurrent/persistent ovary, fallopian tube or primary peritoneal clear-cell carcinoma, harbouring PIK3CA ^2^ hotspot mutations (*n* = 86)	CYH33 monotherapy	ORR ^3^ in patients with PI3KCA hotspot mutation	PFS ^4^, OS ^5^, biomarker alterations impacting PI3K pathway	14 September 2021	NCT05043922
**Dose Escalation of RMC-5552 Monotherapy in Relapsed/Refractory Solid Tumour**	RMC-5552	mTORC1	Phase I, single arm	Relapsed or advanced refractory solid tumours. (*n* = 108)	RMC-55 monotherapy with dose escalation phase and dose expansion phase (stratified by mTOR pathway aberrations)	AEs ^6^, DLTs ^7^	PKs ^8^, ORR (overall response rate), DOR ^9^	1 March 2021	NCT04774952
**A Study Evaluating the Efficacy and Safety of Biomarker-Driven Therapies in Patients with Persistent or Recurrent Rare Epithelial Ovarian Tumours (BOUQUET)**	Ipatasertibinavolisib	AKT PI3K	Phase II, platform study	Persistent or recurrent rare ovarian cancer (*n* = 400)	Stratification into 8 arms depending on biomarker expression: (1) ipatasertib + paclitaxel, (2) cobimetinib, (3) trastuzumab emtansine, (4) atezolizumab + bevacizumab, (5) giredestrant + abemaciclib, (6) inavolisib + palbociclib, (7) inavolisib + palbociclib + letrozole, and (8) inavolisib + olaparib.	ORR ^3^	DOR, DCR ^10^, PFS, OS, and AEs.	18 June 2021	NCT04931342
**Testing the Addition of Ipatasertib to the Usual Chemotherapy Treatment (Paclitaxel and Carboplatin) for Stage III or IV Epithelial Ovarian Cancer**	Ipatasertib	AKT	Single-arm phase I/Ib trial	High-grade serous ovarian cancer, and endometrioid adenocarcinoma. (*n* = 24)	Carboplatin + paclitaxel for up to 3 cycles + ipatasertib until 24 h before surgery	DLT ^11^ in dose escalation and dose expansion phase, AEs	Tumour response	14 March 2022	NCT05276973
**SMMART Adaptive Clinical Treatment (ACT) Trial**	Alpelisib	PI3K	Early phase 1, open-label, multiple arm	Advanced and recurrent malignant solid neoplasm ovarian, pancreatic, prostate, sarcoma breast	Tumour mutational screening and blood collection followed by assignment to one of the trial arms	Proportion of participants who receive an ACT therapy based an ACT Tumour Board recommendation	AEs, ORR, PFS, OS, DSP, toxicity, and tolerability, DSS ^12^	14 February 2022	NCT05238831
**Phase I Trial of VS-6766 Alone and in Combination with Everolimus (RAF/MEK)**	Everolimus	mTORC1	Phase I, non-randomized	Solid tumours or multiple myeloma refractory to conventional treatment (*n* = 104)	3 + 3 dose escalation design with an intermittent once a week schedule A, and if tolerated, twice a week schedule B for VS-6766 in combination with everolimus; the dose expansion cohort will include *KRAS* mutant lung cancer	Recommended phase 2 dose (R2PD), for VS-6766, alone and in combination with everolimus, toxicity profile of VS-6766 alone and in combination with everolimus	PKs of VS-6766, tumour response of VS-6766, as a single agent and also in combination with everolimus	3 April 2015	NCT02407509
**First-in-Human Study of STX-478 as Monotherapy and in Combination with Other Antineoplastic Agents in Participants with Advanced Solid Tumours**	STX-478	PI3K	Multipart, open-label, phase 1/2 study, sequential assignment	Advanced solid tumours breast cancer gynaecologic cancer HNSCC solid tumours	Part 1 will evaluate STX-478 as monotherapy in participants with advanced solid tumours and breast cancer; part 2 will evaluate STX-478 therapy as combination therapy with fulvestrant in participants with breast cancer	DLT, PKs, ORR, AEs, change in cDNA levels. and glucose metabolism biomarkers	No secondary outcome measures	14 March 2023	NCT05768139
**Signal TrAnsduction Pathway Activity Analysis in OVarian cancER (STAPOVER)**	Everolimus	mTORC1	Phase II, phase III, non-randomized study.	Recurrent and refractory ovarian cancer	Stratified by functional signal transduction pathway (STP): ER (oestrogen receptors) active tumours, AR (Androgen receptors) active tumours, PI3K active tumours, HH and/or PI3K active tumours.	PFS	Proportion of patients with an actionable active pathway for which targeted therapy is recommended in relation to the number of patients who underwent a biopsy, proportion of patients who receive matched targeted therapy in relation to the number of patients included in each study arm, BOR, one-year survival, OS, predictive value of STA-analysis results on matched targeted therapy response. side effects, health-related quality of lifecost-effectiveness, change in pathway activity score after disease progression compared to pathway activity score before start of matched therapy	8 March 2018	NCT03458221
**A Study to Evaluate the Safety and Tolerability of TOS-358 in Adults with Select Solid Tumours**	TOS-358	PI3K	Phase 1, open-label, single arm	Solid tumours, colorectal, gastric, HER2-negative breast cancer, non-small Cell lung cancer, squamous cell carcinoma of head and neck, urothelial carcinoma, cervical cancer, ovarian cancer, endometrial cancer	Part 1 (multiple ascending doses, locally advanced, recurrent or metastatic select solid tumours with PIK3CA mutation per local assessment; part 2 (RP2D determined in part 1)	Rate of dose-limiting toxicities (DLTs), incidence and severity of adverse events (AEs) and specific laboratory abnormalities graded according to NCI CTCAE v5	No secondary outcome measures	13 January 2023	NCT05683418
**Targeted Therapy Directed by Genetic Testing in Treating Patients with Locally Advanced or Advanced Solid Tumours, The ComboMATCH Screening Trial**	Alpelisib	PI3K	Randomized, open-label, phase II.	Locally advanced or advanced solid tumours, advanced Malignant Solid Breast Cancer Endometrial Carcinoma Fallopian Tube Carcinoma Ovarian Carcinoma Primary Peritoneal Carcinoma	Tumour mutational screening and assignment to 1 of 20 treatment subprotocols.	Accrual, assignment and enrolment to the trial.	Rate of positive outcomes within the treatment trial defined cohorts Concordance between whole exome sequencing (WES) and results from the Designated Laboratory (DL)	3 October 2022	NCT05564377

^1^ PI3K = phosphoinositide 3-kinase, ^2^ PIK3CA = phosphatidylinositol-4,5-bisphosphate 3-kinase catalytic subunit alpha, ^3^ ORR = objective response rate, ^4^ PFS = progression-free survival, ^5^ OS = overall survival, ^6^ AEs = adverse events, ^7^ DLTs = dose-limiting toxicities, ^8^ PKs = pharmacokinetics, ^9^ DOR = duration of response, ^10^ DCR = disease control rate, ^11^ DLT = dose-limiting toxicity, ^12^ DSS = disease-specific survival.

## Data Availability

No extra data is generated in this review article.
